# Treating Bipolar II Depression: a preliminary open-label investigation of a five-day, accelerated repetitive Transcranial Magnetic Stimulation (rTMS) protocol

**DOI:** 10.1192/j.eurpsy.2025.1050

**Published:** 2025-08-26

**Authors:** C. Cavallotto, G. d’Andrea, A. D’Attilio, G. Mammarella, O. Di Marco, L. P. Padula, L. De Risio, F. Zoratto, G. Di Lorenzo, M. Pettorruso, G. Martinotti

**Affiliations:** 1 Department of Neurosciences, Imaging and Clinical Sciences, Università degli Studi G. D’Annunzio; 2 Department of Mental Health, ASL 2 Abruzzo Lanciano-Vasto-Chieti, Chieti; 3 Department of Mental Health and Addiction, ASL Roma 5; 4 Centre for Behavioural Sciences and Mental Health, Istituto Superiore di Sanità; 5 Department of Systems Medicine, University of Rome Tor Vergata, Roma, Italy; 6 Psychopharmacology, Drug Misuse and Novel Psychoactive Substances Research Unit, School of Life and Medical Sciences, University of Hertfordshire, Hatfield, United Kingdom

## Abstract

**Introduction:**

Bipolar disorder is a complex psychiatric condition marked by severe mood swings, including prolonged depressive episodes that constitute approximately 50% of the illness duration. While there is substantial evidence supporting interventions for manic and hypomanic episodes, therapeutic options for bipolar depression remain insufficient and often ineffective. Accelerated repetitive transcranial magnetic stimulation (arTMS) has emerged as a promising treatment modality with the potential to address these gaps. arTMS offers rapid antidepressant effects comparable to traditional repetitive transcranial magnetic stimulation (rTMS) protocols while maintaining a favorable safety and tolerability profile.

**Objectives:**

This prospective, open-label, multicenter study investigates the efficacy and safety of arTMS in treating bipolar II disorder during depressive phases.

**Methods:**

The study enrolled 20 patients who underwent a five-day, four-times-daily arTMS protocol targeting the left dorsolateral prefrontal cortex (DLPFC). The primary outcome measure was the Montgomery–Åsberg Depression Rating Scale (MADRS), assessed at baseline, immediately post-treatment, and at one- and three-month follow-ups. Secondary outcomes included safety and tolerability, with a focus on the risk of manic or hypomanic switches as measured by the Young Mania Rating Scale (YMRS).

**Results:**

Results indicated a significant reduction in MADRS scores from baseline to immediately post-treatment, with a mean difference of -9.80 (Cohen’s d=1.065, p<0.001). Continued improvements were observed at one month (-15.60, d=1.695, p<0.001) and three months (-19.70, d=2.140, p<0.001), with response rates increasing from 15% immediately after treatment to 60% at three months, and remission rates rising from 5% to 55% over the same period. Importantly, arTMS did not result in any significant increase in YMRS scores, indicating no emergence of manic symptoms, and no hypomanic switches were reported.

**Conclusions:**

The findings underscore the rapid onset and sustained effectiveness of arTMS for bipolar depression, with improvements observed immediately after treatment and continuing over the subsequent months. The progressive rise in response and remission rates suggests that therapeutic benefits may become more pronounced over time, highlighting the importance of considering delayed treatment responses. Moreover, the lack of adverse effects on mood polarity supports arTMS as a safer alternative compared to traditional pharmacological treatments, which are often associated with a risk of manic episodes. Future research should include larger, randomized, sham-controlled trials to validate these findings and further explore the neurobiological mechanisms underlying arTMS’s rapid antidepressant effects.

**Disclosure of Interest:**

None Declared

